# Women who have not utilized health Service for Delivery in Nigeria: who are they and where do they live?

**DOI:** 10.1186/s12884-019-2242-6

**Published:** 2019-03-13

**Authors:** Sulaimon T. Adedokun, Olalekan A. Uthman

**Affiliations:** 1 Department of Demography and Social Statistics, Obafemi Awolowo University, Ile-Ife, Nigeria; 2 Warwick-Centre for Applied Health Research and Delivery (WCAHRD), Division of Health Sciences, University of Warwick Medical School, Coventry, UK; 3 Centre for Evidence-based Health Care, Faculty of Medicine and Health Sciences, Stellenbosch University, Cape Town, South Africa

**Keywords:** Delivery, Health, Maternal, Service, Non-utilization, Facility

## Abstract

**Background:**

Health facility delivery has been described as one of the major contributors to improved maternal and child health outcomes. In sub-Saharan Africa where 66% of the global maternal mortality occurred, only 56% of all births take place in health facility. This study examined the individual and contextual predictors of non-use of health service for delivery in Nigeria where less than 40% births occur in health facility.

**Methods:**

Data from 2013 Nigeria Demographic and Health Survey (DHS) involving 20,192 women who had delivery within 5 years of the survey were used in the study. Multilevel multivariable logistics regression models which had the structure of non-use of health service for delivery defined at individual, community and state levels were applied in the analysis. Spatial analysis was also used to capture the locations where the phenomenon is prevalent in the country.

**Results:**

About 62% of the women did not utilize health service during delivery. More than three-quarter of those with no education and 92% of those who did not attend antenatal clinic during pregnancy never utilized health service for delivery. The odds of non-use of health service during delivery increased for women who had no education, from poor households, aged 25–34 years, unmarried, never attended antenatal clinic, experienced difficulty getting to health facility and lived in the most socioeconomically disadvantaged communities and states.

**Conclusions:**

This study has demonstrated that non-utilization of health service for delivery is influenced by individual, community and state level factors, with substantial proportions of women not utilizing such service residing in the northern region of Nigeria. Each level should be adequately considered in the design of the appropriate interventions.

## Background

Health facility delivery has been described as one of the major contributors to improved maternal and child health outcomes [1]. It provides access to appropriate equipment and drugs, skilled attendants and immediate referral to a higher facility [2]. Proportions of health facility delivery vary across continents and regions. While 9 in every 10 births take place in health facility in Europe, Central and East Asia, the Pacific, Latin America and the Caribbean, only 56% of all births occur in health facility in sub-Saharan Africa [2]. This average performance in respect of facility delivery has reflected in the maternal mortality records in the region. As at 2015, maternal mortality rate in sub-Saharan Africa was 546 per 100,000 live births, accounting for 66% of the global maternal deaths [3]. However, a number of steps have been taken in order to increase facility delivery in the region. Such steps include (i) increase in the number of skilled attendants (ii) identifying and tackling the barriers which make it difficult for women to reach the health facility (iii) community engagement where health workers sensitise women on the importance of facility delivery (iv) facilitating change in the norm on home delivery and (v) imposition of fines by government on home delivery [1].

The situation of maternal health in Nigeria aligns with that of sub-Saharan Africa as a whole. The country’s maternal mortality ratio of 576 deaths per 100,000 live births calls for concern [4]. In fact, world records indicate that one-third of the global maternal deaths occurred in Nigeria and India [3]. Since complications resulting from pregnancy contribute substantially to these maternal deaths, strategies have been adopted at different times to improve pregnancy and delivery care. One of such strategies is the SURE-P maternal and child health programme which has been designed to ensure not only access to maternal health services but also the quality of maternal health care [1]. The programme is premised on ensuring adequate staff at facility and renovating existing facility; ensuring adequate availability of essential drugs, equipment and materials; reducing the financial burden for women in respect of attendance during antenatal care, delivery at facility and postnatal care and; increasing awareness at community level through the collaboration of health workers and leadership committees [1]. The Nigeria States Health Programme Investment Project is another programme which provides finance for maternal and child health (MCH) services. It supports health facilities through provision of funds for operational costs, drugs, maintenance and repair and incentives to health workers [5].

In spite of the enormous resources committed towards achieving these objectives, only 36% of births are delivered in health facility in Nigeria [4]. Studies have been conducted to explain the factors that are responsible for the poor health service utilization for delivery in the country. While some studies attributed this to education, age, residence, employment status and household wealth [6–12], others emphasise parity, distance to facility, cultural factors and attitude of staff at facility [13–15]. Meanwhile, most of these studies related such factors to utilization with little emphasis on non-utilization of such services. Even the few studies that examined factors affecting non-utilization of health services for delivery considered mainly non-contextual factors. In order to have a robust explanation for non-utilization of health services, there is a need for a study that would involve not only the individual-level factors but also factors at higher levels. This study aims at filling these gaps by developing a three-level model of non-utilization of maternal health service for delivery defined at individual, community and state levels in Nigeria. In addition, the study provides a spatial analysis of the phenomenon in such a way that the different locations where the problem is prominent have been adequately captured.

## Methods

### Study design

Analyses in this study were done using the 2013 Nigeria Demographic and Health Survey (NDHS) data set. The survey is cross-sectional, population-based and provides information on population and health characteristics.

### Sampling technique

A multi-stage cluster sampling method was used in the 2013 NDHS. The country was categorised into 37 units which included all the 36 states and the Federal Capital Territory (FCT), Abuja. A total of 896 communities (clusters) were selected from these states using the primary sampling unit (PSU) of the 2006 population and census enumeration areas. The chosen communities were further disaggregated into enumeration areas in which 532 were created in the rural areas while the urban areas had 372. Households were then randomly selected from the enumeration areas. A total of 40,680 households were finally chosen with 23,940 and 16,740 in the rural and urban areas respectively.

### Data collection

Details of data collection have been published elsewhere [16]. Questionnaires were used to obtain information from women aged 15–49 years through household interviews. Such women were asked to provide information about their socioeconomic characteristics, reproduction, breastfeeding practice, domestic violence, child care practice and health service use during pregnancy, delivery and postnatal period.

### Outcome variable

The study focused on women aged 15–49 years who gave birth to children within five years of the survey. Women who delivered at health facility, either private or public, were defined as utilizing health service for delivery while those who delivered elsewhere were defined as not utilizing health service for delivery. The former was subsequently defined as a binary variable assuming the value of 1 while the latter assumed the value of 0.

### Explanatory variables

#### Individual-level factors

The variables that constituted individual level factors include: age, education, household wealth index, occupation, marital status, mass media exposure and antenatal care attendance. Age was defined as 15–24 years, 25–34 years and 35+ years. Education was expressed as no education, primary, secondary or higher education. Since participants’ response on income in developing countries is often characterised with inaccuracy, household wealth index was used as a measure for wealth status. This wealth index was obtained by considering the ownership of household commodities such as television, radio, type of roofing/floor, water source and dwelling features. This approach, which is based on principal component analysis, has been used by the World Bank to define household poverty level [17, 18]. Although DHS presented the wealth index in five quintiles, we regrouped these quintiles into three tertiles (poor, middle and rich). Occupation was grouped into working and not working. Marital status has two categories: ever married and never married. Mass media exposure was defined as ever exposed for those who have access to at least one of newspaper, radio or television, and never exposed for those who have access to none. Antenatal care attendance was grouped into women who never attended, those who had less than 4 visits and those who had 4 or more visits.

#### Community-level factors

The following factors were considered at community level: place of residence (rural or urban), getting to health facility (being a problem or not a problem), ethnicity diversity index and socioeconomic status. Socioeconomic status was derived from the proportions of individuals who are unemployed, illiterate and poor. This was then categorised into tertile 1 (least disadvantaged), tertile 2 and tertile 3 (most disadvantaged). Ethnicity diversity index was a variable obtained using the formula:

$$ \mathrm{Ethnic}\ \mathrm{diversity}\ \mathrm{index}=1-{\sum}_{\mathrm{i}=1}^{\mathrm{n}}{\left[\frac{x_i}{y}\right]}^2 $$

Where: *x*_*i*_ = population of ethnic group i of the area, y = total population of the area, n = number of ethnic groups in the area.

It reflects the spread of ethnic groups by calculating values from 0 to 1. This is then multiplied by 100 to arrive at the diversity [19]. The higher the value the more widespread the community. While an index of 0 indicates a community is mono-ethnic in nature, an index of 1 shows that such a community is multi-ethnic in nature.

#### State-level factors

The state-level factor was derived from the proportions of individuals in the state who are unemployed, illiterate and poor. This was then categorised into tertile 1 (least disadvantaged), tertile 2 and tertile 3 (most disadvantaged).

### Statistical analyses

#### Descriptive statistics

In the descriptive analysis which involved the use of Chi-Square test, the independent variables at each level were presented using numbers and percentages.

#### Modelling approaches

A three-level binomial regression model consisting of individual, community and state was constructed due to the hierarchical nature of the data set. Four models were thereafter specified. In the first model which was specified in order to decompose the amount of variance found between the community and state levels, no explanatory variables were included. Individual and community level variables were included in the second and third models respectively. The last model contained the state level variables in addition to the variables from individual and community levels.

#### Fixed effects (measures of association)

The results of fixed effects were presented in terms of odds ratios (OR) together with their 95% credible intervals (CrI).

#### Random effects (measures of variation)

Results of random effects were presented using three measures: the intra-cluster correlation (ICC), variance partition coefficient (VPC) and median odds ratio (MOR). MOR measures cluster heterogeneity that remains unexplained. Information on the procedure for computing MOR has been published elsewhere [20, 21].

#### Model fit and specification

While goodness of fit of the model was checked using Bayesian Deviance Information Criterion (DIC), multicollinearity was assessed by applying Variance Inflation Factor (VIF). MLwiN 2.35 [22] calling Stata Statistical Software version 14 (Stata, 2015) was used to carry out all the multilevel modelling operations. Also, the operation involved Markov Chain Monte Carlo (MCMC) estimation [23].

#### Spatial analysis

Results of the spatial analysis were presented using percentile map, excess risk map, global spatial autocorrelation (Moran’s I) map and funnel plot. Percentile map showed the prevalence of non-use of health service for delivery in four categories: low prevalence (3–10%); moderate prevalence (10–25%); high prevalence (25–45%) and; very high prevalence (45–70%). The excess risk map revealed the expected number of women versus the observed number of women who did not utilize health service for delivery. States with value greater than 2 are considered to have excess risk above the expected while states with value less than 2 are considered to have excess risk less than the expected. Global spatial analysis (Moran’s I) presented the distribution of non-use of health facility for delivery in four groups:

High-high: this indicates high rate of non-use of health service for delivery in a particular state with the adjoining states experiencing high rates of non-facility delivery.

Low-low: low rate of non-use of health facility for delivery in a state with the adjoining states having low rates as well.

High-low: high rate of non-use of health service for delivery in a state with the adjoining states experiencing low rates of non-facility delivery.

Not significant: this group involves states with values that are not statistically significant.

The spatial analysis was performed by applying the exploratory spatial data analysis (ESDA) method using GeoDa software [24].

## Results

### Sample characteristics

Table 1 shows the summary of the respondents’ characteristics. The analysis involved 20,192 women aged 15–49 years (level 1), nested within 896 communities (level 2) and from 37 states (level 3) in Nigeria. About 62% of the women did not utilize health service for delivery. Among these women, more than three-quarter of those with no education (87%) and 91% of those from poor households never utilized health service for delivery. Significant proportion of women who did not attend antenatal clinic (91.6%) never utilized health service for delivery. While three-quarter of women from the rural area (75.4%) did not utilize health service for delivery, about 78% of those who complained that getting to health facility was a problem did not deliver at such facility. At least 8 in every 10 women who lived in the most socioeconomically disadvantaged states and communities did not utilize health service during delivery.

**Table 1:** Health service utilization for delivery at different levels of independent variables

Variable	Utilized healthcare service for delivery			
	Yes	No	Total	*p*-value
	*N* (%)	(%)	N (%)	
Individual-level factors	7720 (38.2)	12,472 (61.8)	20,192 (100.0)	
Age				
15–24	1676 (32.4)	3504 (67.6)	5180 (100.0)	
25–34	3848 (40.7)	5616 (59.3)	9464 (100.0)	
35+	2196 (39.6)	3352 (60.40	5548 (100.0)	< 0.001
Education				
No education	1171 (12.8)	8000 (87.2)	9171 (100.0)	
Primary	1713 (41.7)	2400 (58.3)	4113 (100.0)	
Secondary/higher	4836 (70.0)	2072 (30.0)	6908 (100.0)	< 0.001
Household wealth index				
Poor	642 (9.5)	6089 (90.5)	6731 (100.0)	
Middle	2327 (34.6)	4405 (65.4)	6732 (100.0)	
Rich	4751 (70.6)	1978 (29.4)	6729 (100.0)	< 0.001
Occupation				
Not working	1848 (29.3)	4461 (70.7)	6309 (100.0)	
Working	5872 (42.3)	8011 (57.7)	13,883 (100.0)	< 0.001
Marital status				
Never married	247 (45.9)	291 (54.1)	538 (100.0)	
Ever married	7473 (38.0)	12,181 (62.0)	19,654 (100.0)	< 0.001
Mass media exposure				
Never exposed	1160 (16.9)	5696 (83.1)	6856 (100.0)	
Exposed	6560 (49.2)	6776 (50.8)	13,336 (100.0)	< 0.001
Antenatal care attendance				
Never attended	606 (8.4)	6596 (91.6)	7202 (100.0)	
< 4 visits	697 (28.1)	1786 (71.9)	2483 (100.0)	
4 or more visits	6417 (61.1)	4090 (38.9)	10,507 (100.0)	< 0.001
Community-level factors				
Residence				
Urban	4420 (65.1)	2370 (34.9)	6790 (100.0)	
Rural	3300 (24.6)	10,102 (75.4)	13,402 (100.0)	< 0.001
Getting to health facility				
Not a problem	6293 (45.7)	7491 (54.3)	13,784 (100.0)	
A problem	1427 (22.3)	4981 (77.7)	6408 (100.0)	< 0.001
Ethnicity diversity index			2.50 (2.81)	
Socioeconomic disadvantage				
Tertile 1 (least disadvantaged)	4603 (68.3)	2139 (31.7)	6742 (100.0)	
Tertile 2	2663 (39.5)	4078 (60.5)	6741 (100.0)	
Tertile 3 (most disadvantaged)	454 (6.8)	6255 (93.2)	6709 (100.0)	< 0.001
State-level factors				
Socioeconomic disadvantage				
Tertile 1 (least disadvantaged)	4550 (64.4)	2520 (35.6)	7070 (100.0)	
Tertile 2	2441 (33.9)	4766 (66.1)	7207 (100.0)	
Tertile 3 (most disadvantaged)	729 (12.3)	5186 (87.7)	5915 (100.0)	< 0.001

### Measures of association (fixed effects)

Results of the different models are shown in Table 2. In the fully adjusted model (model 4), age, education, household wealth, marital status, antenatal care attendance, residence, having problem getting to health facility and socioeconomic status at both state and community levels were significantly associated with non-utilization of health service for delivery. Women aged 25–34 are 14% more likely to not utilize health service during delivery compared with women aged 35 years and above. Women with no education are 138% more likely to not utilize health service for delivery compared with those who have secondary or higher education. The odds of not utilizing health service during delivery increased by 144% for women from poor households compared with their counterparts from rich households. Unmarried women have higher likelihood of not delivering at health facility as the odds reduced by 36% for married women. Women who never attended antenatal clinic are 531% more likely to not utilize health service during delivery compared with women who attended antenatal clinic 4 or more times. The chances of not utilizing health service during delivery increased for women who lived in the rural area (OR = 1.81; 95% CrI = 1.54–2.12), experienced problems getting to health facility (OR = 1.28; 95% CrI = 1.15–1.44) and lived in the most socioeconomically disadvantaged communities (OR = 2.31; 95% CrI = 1.68–3.21) and states (OR = 4.21; 95% CrI = 1.85–7.89).

**Table 2:** Multilevel logistic regression models of factors associated with non-utilization of health service for delivery

Variable	Model 1^a^	Model 2^b^	Model 3^c^	Model 4^d^
	aOR (CrI)	aOR (CrI)	aOR (CrI)	aOR (CrI)
Individual-level factors				
Age				
15–24		1.03 (0.89–1.18)	0.99 (0.86–1.13)	0.98 (0.86–1.10)
25–34		1.16 (1.03–1.29)	1.15 (1.04–1.28)	1.14 (1.03–1.26)
35+		1 (reference)	1 (reference)	1 (reference)
Education				
No education		2.64 (2.28–3.03)	2.42 (2.09–2.75)	2.38 (2.07–2.73)
Primary		1.89 (1.68–2.12)	1.84 (1.64–2.04)	1.84 (1.63–2.05)
Secondary/higher		1 (reference)	1 (reference)	1 (reference)
Household wealth index				
Poor		3.42 (2.84–4.06)	2.44 (2.03–2.92)	2.44 (1.98–2.86)
Middle		2.06 (1.82–2.32)	1.72 (1.49–1.95)	1.71 (1.50–1.92)
Rich		1 (reference)	1 (reference)	1 (reference)
Occupation				
Not working		1 (reference)	1 (reference)	1 (reference)
Working		0.99 (0.90–1.09)	1.006 (0.91–1.10)	0.99 (0.90–1.10)
Marital status				
Never married		1 (reference)	1 (reference)	1 (reference)
Ever married		0.67 (0.53–0.87)	0.63 (0.47–0.83)	0.64 (0.49–0.80)
Mass media exposure				
Never exposed		1 (reference)	1 (reference)	1 (reference)
Exposed		0.89 (0.79–0.99)	0.92 (0.82–1.04)	0.92 (0.81–1.03)
Antenatal care attendance				
Never attended		6.75 (5.95–7.59)	6.34 (5.62–7.13)	6.31 (5.56–7.12)
< 4 visits		1.98 (1.75–2.23)	1.94 (1.72–2.18)	1.93 (1.69–2.17)
4 or more visits		1 (reference)	1 (reference)	1 (reference)
Community-level factors				
Residence				
Urban			1 (reference)	1 (reference)
Rural			1.79 (1.42–2.15)	1.81 (1.54–2.12)
Getting to health facility				
Not a problem			1 (reference)	1 (reference)
A problem			1.27 (1.14–1.40)	1.28 (1.15–1.44)
Ethnicity diversity index			0.97 (0.95–1.006)	0.97 (0.94–1.003)
Socioeconomic disadvantage				
Tertile 1 (least disadvantaged)			1 (reference)	1 (reference)
Tertile 2			1.39 (1.13–1.74)	1.32 (1.07–1.63)
Tertile 3 (most disadvantaged)			2.59 (1.77–3.68)	2.31 (1.68–3.21)
State-level factors				
Socioeconomic disadvantage				
Tertile 1 (least disadvantaged)				1 (reference)
Tertile 2				1.40 (0.75–3.23)
Tertile 3 (most disadvantaged)				4.21 (1.85–7.89)
Measures of variation				
State level				
Variance (SE)	3.954 (2.388–6.563)	1.294 (0.777–2.118)	0.875 (0.507–1.438)	0.741 (0.436–1.233)
Explained variation (%)	Reference	67.3	77.9	81.2
ICC (%)	43.11	24.40	18.15	15.83
MOR	6.66	2.96	2.44	2.27
Community level				
Variance (SE)	1.926 (1.674–2.203)	0.718 (0.603–0.845)	0.657 (0.541–0.773)	0.651 (0.540–0.774)
Explained variation (%)	Reference	62.7	65.9	66.2
ICC (%)	64.11	37.93	31.75	29.72
MOR	3.78	2.24	2.17	2.16
Model fit statistics				
Bayesian DIC	16,555	15,176	15,097	15,098

^a^Model 1 is the empty model, a baseline model with no independent variable

^b^Model 2 is adjusted for age, education, household wealth index, occupation, marital status, mass media exposure, and antenatal care attendance

^c^Model 3 is additionally adjusted for residence, getting to health facility, ethnicity diversity index and community socioeconomic factors

^d^Model 4 is additionally adjusted for state socioeconomic factors

Abbreviations: SE; standard error, DIC; deviation information criterion, CrI; credible interval, ICC; intra-cluster correlation, MOR; median odds ratio

### Measures of variation (random effects)

As shown in Table 2 in the unconditional model (model 1), there was a significant variation in the odds of non-utilization of health service for delivery across the states (*σ*^2^= 3.95; 95% CrI = 2.39–6.50) and across the communities (*σ*^2^= 1.93; 95% CrI = 1.67–2.20). The intra-state and intra-community correlation coefficients reveal that 43.1 and 64.1% of the variance in odds of not utilizing health service during delivery are attributed to state and community-level factors respectively. The results from the MOR in the fully adjusted model (model 4) reflect the significant contributions of community and state-level factors to maternal health service utilization. If a woman moved to another state or community with a higher probability of non-utilization of health service for delivery, the likelihood of not delivering at health facility would increase by 2.27 and 2.16 times respectively.

### Spatial distribution and analysis

**Fig. 1:**
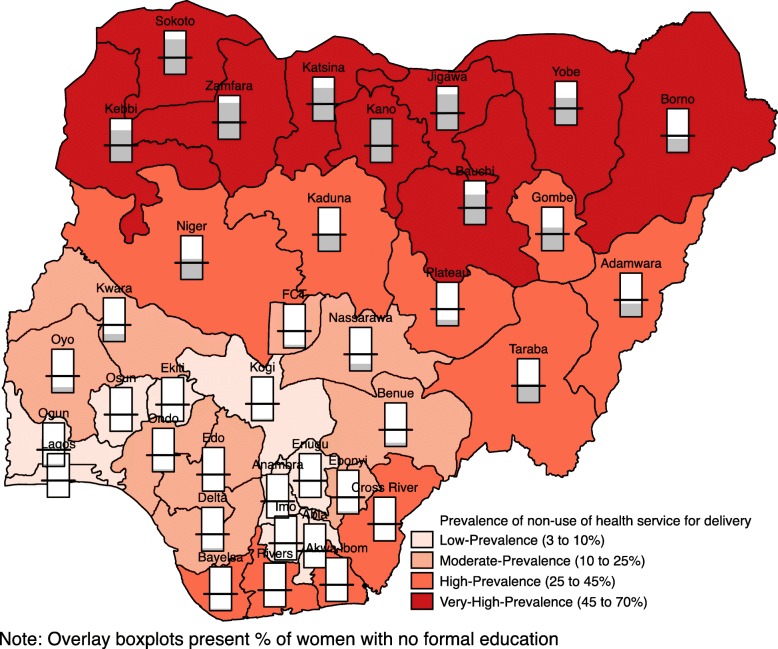
Percentile map showing the prevalence of non-use of health service for delivery and level of illiteracy among mothers

The prevalence of non-utilization of health service for delivery is shown in Fig. 1. Nine states have low prevalence (3–10%), 9 states also have moderate prevalence (10–25%), 10 states have high prevalence (25–45%) and 9 states are considered to have a very high prevalence (45–70%). States with low prevalence are found mainly in the southern region of the country and they are Kogi, Ekiti, Osun, Ogun, Lagos, Enugu, Anambra, Imo and Abia. The moderate prevalence category includes states such as Kwara, Oyo, Ondo, Edo, Delta, Nasarawa, Benue, Ebonyi and the Federal Capital Territory. Adamawa, Taraba, Plateau, Kaduna, Niger, Cross River, Akwa Ibom, Rivers, Bayelsa and Gombe are found in the high prevalence category. The very high prevalence category consists of states in the northern region. Such states include Sokoto, Zamfara, Katsina, Kano, Jigawa, Bauchi, Yobe and Borno. There is also high illiteracy level among mothers in the states with a very high prevalence of non-utilization of health service during delivery.

**Fig. 2:**
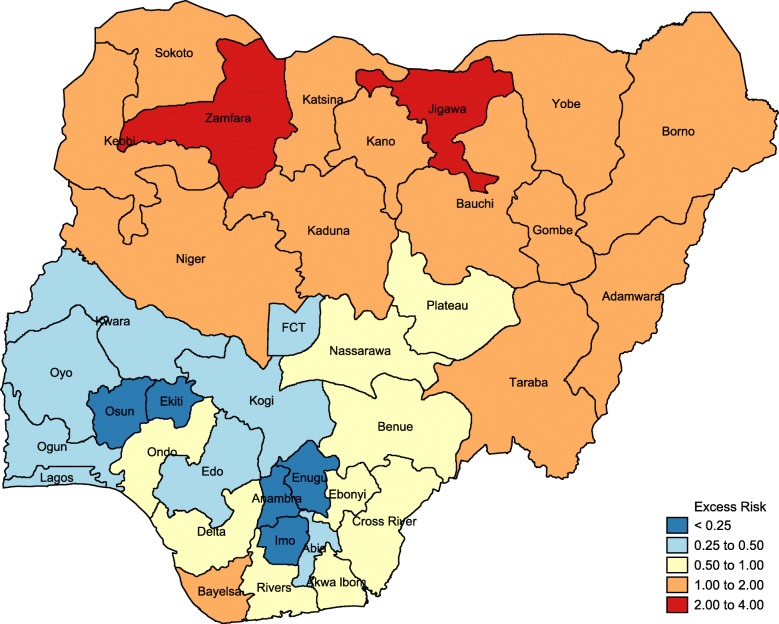
Excess risk map showing non-use of health service for delivery in all states in Nigeria

Results from the excess risk map, which relates expected number of women to the observed number of women who did not utilize health service for delivery, are presented in Fig. 2. The states in red have higher rates of excess risk above the expected (> 2). The two states in this category are Zamfara and Jigawa. There are five states with excess risk extremely lower than the expected (< 0.25). Such states are marked in blue and they include Osun, Ekiti, Enugu, Anambra and Imo.

**Fig. 3:**
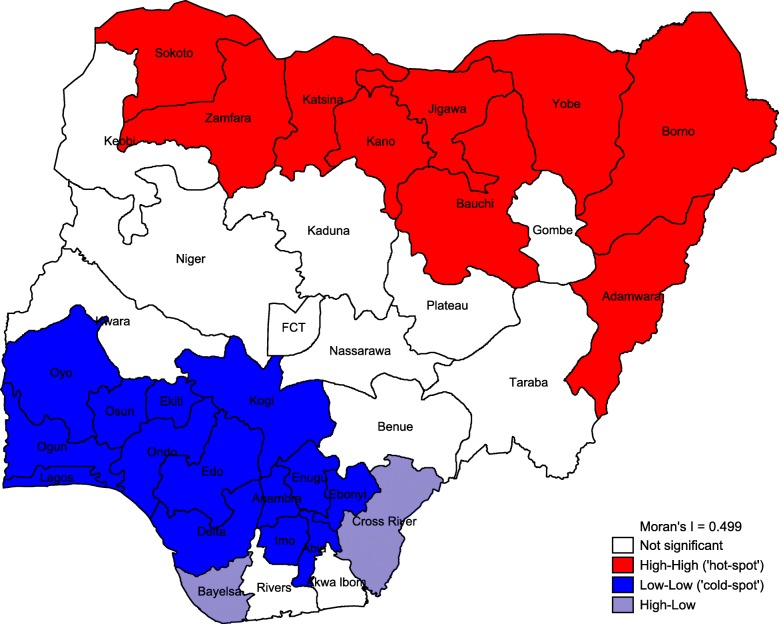
Global spatial autocorrelation map showing non-use of health service for delivery in all states in Nigeria

Results from global spatial autocorrelation (Local Moran’s I) are presented in Fig. 3. States with low-low category are marked in blue. Such states are described as the cold-spot with low percentage of women who did not utilize health service during delivery. Such states include Oyo, Ogun, Osun, Lagos, Ekiti, Ondo and Kogi. Others are Edo, Delta, Anambra, Enugu, Ebonyi, Imo and Abia. These states, all located in the southern region of the country, are surrounded by other states that have similar status of being described as cold-spot. The high-high category marked in red depicts states with high percentage of women who did not utilize health service for delivery. This category comprises Sokoto, Zamfara, Katsina, Kano and Jigawa. Others include Bauchi, Yobe, Borno and Adamawa. Such states are surrounded by other states that have similar status of being described as hot-spot. All the states in this category are located in northern region.

**Fig. 4:**
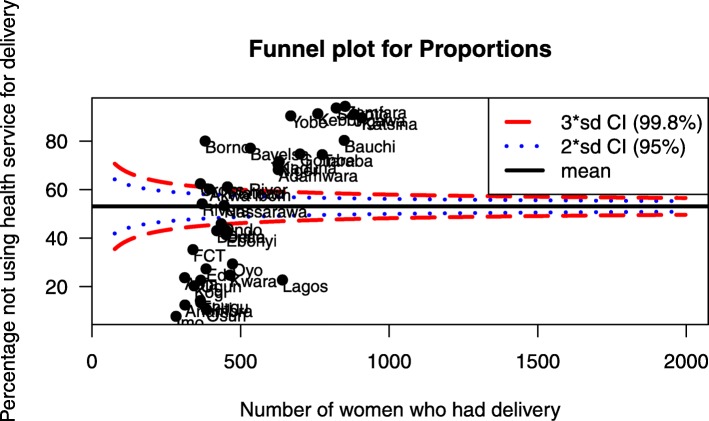
Funnel plot showing proportions of non-facility delivery in Nigeria

Figure 4 presents results from the funnel plot which shows the percentage of women not using health service during delivery against the number of women who had delivery. The diagram shows some states above the average rate, indicating high rates of non-utilization of health service. Such states include Bauchi, Yobe, Zamfara, Katsina, Adamawa and Gombe, among others. Some states are found below the average rate, indicating low rates of non-utilization of health service for delivery. Some of the states in this category are Lagos, Oyo, Osun, Kwara, and Imo, among others.

## Discussion

This study has provided a robust information on the high prevalence of non-utilization of health service for delivery in Nigeria; the factors that precipitate such non-utilization of health service and; the locations where the phenomenon is well pronounced. With 62% of women not making use of health service during delivery, the goal of having a substantial reduction in maternal mortality through improved delivery care may remain unachievable. Such scenario also leads to a waste of resources that have been provided for the implementation of such services. Our study revealed the important roles individual, community and state level factors play in maternal health service utilization. At the individual level, age, education, household wealth, marital status and antenatal care attendance influence maternal health service utilization. Non-use of health service during delivery among middle-aged women (25–34) is higher compared to women in older age category (35+). Although it may be opined that the latter comprises those whose pregnancy is considered risky as a result of their age bracket, which consequently prompts them to seek facility delivery, the former also consists of women who are at the prime of their childbearing period. Such women need to avail themselves of the opportunity of seeking facility delivery in order to avert or address any complications that may arise. Low prevalence of non-utilization of health service among older women could also be attributed to the childbearing experience of such women. These women may have been exposed to the danger of non-facility delivery at one time or the other or had friends or relatives who have been victims of complications during delivery outside health facility. Previous studies have identified age as an important predictor of maternal health service utilization [25–27]. The study also reflected the importance of education in maternal health service use. The less educated a woman becomes, the higher her chances of having non-facility delivery [28–32]. Well educated women are more often than not exposed to vital information about health care utilization, including facility delivery. Such women are likely not to subscribe to some notions in the community which constitute impediments to maternal health service use. One of such notions is the idea that women who experience non-facility delivery are physically strong and as a result should be held in high esteem. Household wealth influences health service use as the probability of non-utilization of health service for delivery increased tremendously among women from poor households. Such women may lack the fund for transportation especially when they live far away from where they could access the health service. In addition, the women may opt for non-facility delivery if they consider the costs of delivery care to be unaffordable. Other studies have also emphasised the impact of poverty on health service use [33–37].

Findings from our study also showed the effect of marital status on maternal health service use. Unmarried women have higher likelihood of not using health service for delivery compared to married women [38]. This may be linked to lack of spousal support on the part of unmarried women which, contrarily, married women enjoy. The role of antenatal care has been emphasised in previous studies [39–41]. Our study revealed that the less the number of antenatal clinic a woman attends, the more her likelihood of not delivering in health facility. Regular attendance of antenatal care affords women different opportunities in respect of benefits they enjoy. Such women enjoy having the condition of their pregnancies properly monitored and they are regularly exposed to health education where emphasis is placed on facility delivery. At the community level, residence, difficulty in getting to health facility and socioeconomic status exert influence on health service utilization for delivery. Rural women are more likely to not use health service for delivery. Unlike women in urban areas, rural women have access to limited number of health facility [42, 43]. Even some of the available ones may lack personnel or equipment that would enhance safe delivery. In some cases, knowledge of the unavailability of the equipment and personnel may discourage pregnant women from utilizing the facility for delivery [44]. Another challenge in the rural area is the proliferation of traditional birth attendants who lack the orthodox techniques of delivery care. Some women may consider their services cheap and readily available [45]. The difficulty experienced in getting to health facility may also discourage women from using health service during delivery. Such difficulty may come in form of lack of transportation due to remoteness of the residence. Some women may eventually deliver at home while in the process of looking for vehicle to convey them to the nearest health facility [44, 46–49]. The more a community is disadvantaged socioeconomically, the higher the likelihood of women in that community to not utilize health service during delivery [50, 51]. The same scenario is depicted at the state level. Being socioeconomically disadvantaged reflects the prevailing condition of a community or state in terms of high rates of unemployment, illiteracy and poverty. The spatial analysis gave a detailed description of the spread of non-utilization of health service during delivery across the 37 units which constitute 36 states and the federal capital of the country. The analysis showed that states with very high prevalence of non-use of health service for delivery are all located in the northern region of the country. There is also high illiteracy level among women in these states which further underscores the correlation between high illiteracy rate and non-facility delivery. By extension, the states that are considered hot-spot and have excess risk above the expected are all found in the north. Contrarily, all the states with low prevalence, excess risk below the expected and in the cold-spot category are located in the south. This reflects the dichotomy in non-utilization of health service for delivery between the north and south in Nigeria.

### Policy implications

With 62% of women not utilizing health service for delivery in Nigeria, it is obvious that much still needs to be done to increase facility delivery. To achieve this, it is important to give special attention to maternal health care in northern region of the country. This region is home to: all the 9 states with a very high prevalence of non-utilization of health service during delivery; the only 2 states with excess risk of non-utilization of health service above the expected level and; all the 9 states that are categorised as hot-spots for non-use of health service for delivery. This indicates that the national rate of 62% would reduce considerably if the situation can be brought under control in the north. In view of this, the federal and state governments have roles to play. Since there is a strong correlation between level of illiteracy and non-use of health service during delivery, with high illiteracy level among women in the north, northern state governments should intensify efforts on increasing the proportion of educated women. This can be achieved by adopting a two-edge approach: encouraging the enrolment of young girls in school and committing more resources to adult education in order to give illiterate women the opportunity of receiving formal education. Also, in the process of providing formal education for the women, regular sessions on health education which emphasises the necessity of health service utilization during pregnancy and delivery should be incorporated. At the federal level, National Health Insurance Scheme (NHIS) could be used to increase maternal health service utilization. NHIS was established to ensure that individuals have access to good health and enjoy a relief from the burden of huge medical bills [52]. It caters for individuals in formal and informal sectors. However, the scheme could be strengthened to give priority to some maternal health components. For instance, the costs of delivery could be subsidised particularly for those in the informal sector such that they would be responsible for 5% of total amount incurred. This would go a long way in encouraging women from poor households to use health service.

There is a need to embark on regular review of programmes and initiatives that have been introduced to improve maternal health care use. Such review should be done for the purpose of assessing not only the effectiveness of the programme as a whole but also effectiveness of the components. Fresh ideas that would enhance utilization should be incorporated. For instance, the Nigeria States Health Programme Investment Project which provides for drugs, operational costs and incentives to health workers could be strengthened to include financial supports for delivery. At the same time, efforts should be made to assess the scope of SURE-P maternal and child health services which, among other things, aimed at reducing financial burden for women in relation to antenatal care, delivery at health facility and postnatal care. The assessment should be tailored towards providing answers to these questions: does the programme cover all the states of the federation? What categories of women are included? What is the magnitude of the subsidy being provided? To what extent is the programme directed at women in northern Nigeria? More resources need to be committed to infrastructural developments by state governments. Construction of roads and other infrastructural facilities particularly in remote areas should be given top priority. This would reduce the difficulty being experienced to reach health facility. The community-health workers partnership on awareness programme should be sustained. Involvement of community and religious leaders in the campaign for the significance of health service utilization for delivery should be emphasised. More so, there is a need for government-community partnership for provision of standby vehicle to convey women experiencing any sign of labour to the nearest health facility. Such partnership may be designed to make the government contribute 60% of the fund for the procurement of the vehicle while the community would cater for the balance. Above all, efforts should be made by governments both at the state and federal levels to improve conditions of people in the socioeconomically disadvantaged communities and states. Programmes aimed at reducing poverty, unemployment and illiteracy should be vigorously pursued.

### Study strengths and weaknesses

Findings from this study have been derived from the analysis of DHS data set. The survey obtained information from women based on self-reports. Such reports are sometimes liable to recall error or bias. In addition, the survey is cross-sectional and as a result, causality could not be established. In spite of these shortcomings, our study is based on nationally representative data set which allows for generalization of results.

## Conclusions

This study revealed that factors influencing non-utilization of health service for delivery in Nigeria operate at individual, community and state levels, with preponderance of women not using health service residing in northern region of the country. Interventions aimed at addressing this problem should be designed in such a way that each level would be adequately considered.

## Abbreviations

aOR: Adjusted Odds Ratio
CrI: Credible Interval
DIC: Deviation Information Criterion
ESDA: Exploratory Spatial Data Analysis
ICC: Intra-Cluster Correlation
MOR: Median Odds Ratio
NDHS: Nigeria Demographic and Health Survey
NHIS: National Health Insurance Scheme
SE: Standard Error
